# Psychobiotics in mental health: insights from human clinical trials via the gut-brain axis

**DOI:** 10.3389/fmicb.2026.1804560

**Published:** 2026-03-27

**Authors:** Natarajan Sisubalan, Periyanaina Kesika, Bhagavathi Sundaram Sivamaruthi, Chaiyavat Chaiyasut

**Affiliations:** 1 PG and Research Department of Botany, Pachaiyappa’s College, Affiliated to University of Madras, Chennai, Tamil Nadu, India; 2 Office of Research Administration, Chiang Mai University, Chiang Mai, Thailand; 3 Innovation Center for Holistic Health, Nutraceuticals, and Cosmeceuticals, Faculty of Pharmacy, Chiang Mai University, Chiang Mai, Thailand

**Keywords:** anxiety, depression, gut-brain axis, probiotics, psychobiotics

## Abstract

Recent research has highlighted the gut-brain axis as a critical modulator of mental health, positioning probiotics as promising psychobiotic interventions for anxiety, stress, depression, and cognitive function. Clinical trials investigating strains such as *Lactobacillus rhamnosus, Bifidobacterium longum, Lactobacillus gasseri*, and multi-strain formulations have demonstrated strain- and context-specific effects on psychological and physiological outcomes. While some studies reported improvements in mood, anxiety, sleep quality, and cognitive performance, others showed limited effects, particularly in healthy populations with low baseline stress levels, reflecting the challenges of translating preclinical findings into clinical applications in humans. Mechanistic evidence suggests that psychobiotics may influence neuroactive metabolites, short-chain fatty acids, *γ*-aminobutyric acid, serotonin, and anti-inflammatory pathways, thereby modulating cognitive and emotional processes. The effects happen dependent on factors such as dosage, strain specificity, delivery method, baseline stress or symptom levels, and co-administration with conventional treatments. Collectively, these findings underscore the potential of targeted psychobiotics to enhance mental well-being and support stress resilience, while highlighting the need for carefully designed clinical trials to clarify efficacy and mechanisms.

## Graphical abstract

**Figure:**
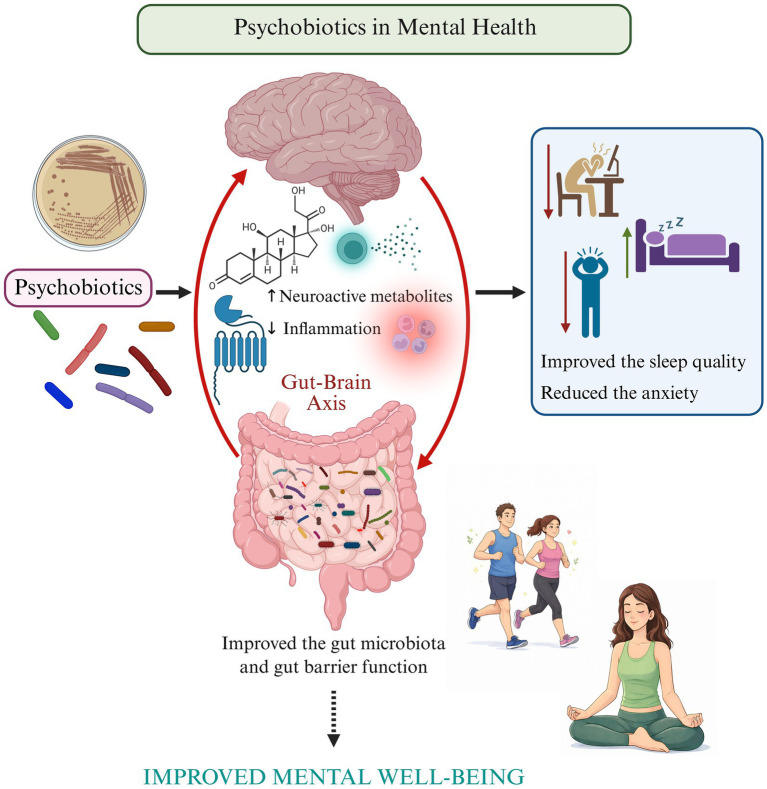
Psychobiotics regulate the gut–brain axis through microbial metabolites and anti-inflammatory effects, improving gut health and contributing to reduced anxiety, better sleep, and enhanced mental well-being.

## 1 Introduction

The concept that human health is closely linked to gut function dates to antiquity, as reflected in Hippocrates’ statement, “Death lies in the bowel,” which emphasizes the central role of gut health in overall well-being (Bistoletti et al., 2020). The gastrointestinal tract is the primary site of nutrient and bioactive molecule absorption, enabling extensive bidirectional communication with multiple organ systems. Among these interactions, the relationship between the gut and the brain is particularly prominent and is mediated through interconnected signaling pathways collectively known as the gut-brain axis (Ahmed et al., 2022). Growing interest in this field has positioned the gut-brain axis as a focal point of body–brain research, with the gut microbiota emerging as a key target of clinical and therapeutic relevance (Dinan et al., 2013). The microbiota-gut-brain axis (MGBA) encompasses a complex communication network involving the gastrointestinal tract, resident microbial communities, and the peripheral and central nervous systems (Mörkl et al., 2020).

Multiple biological mechanisms contribute to MGBA signaling, including immune modulation (Park et al., 2025), neural communication via the autonomic nervous system (O'Riordan et al., 2025), particularly the vagus nerve (Cryan et al., 2019), microbial metabolite production such as bile acids, choline, and short-chain fatty acids, and the synthesis and metabolism of neurotransmitters, including *γ*-aminobutyric acid (GABA), serotonin, dopamine, noradrenaline, and acetylcholine (Mörkl et al., 2020; Chen et al., 2021; O'Riordan et al., 2022; Tingler et al., 2025; Wang et al., 2026). The psychological functioning is increasingly viewed as an embodied process arising from the integration of brain activity with peripheral physiological systems, in which the microbiome represents a functional component of the human body (Ma et al., 2024). In parallel, lifestyle behaviors such as dietary habits (Aslam et al., 2026), physical activity (Min et al., 2024), and exposure to natural environments (Zhang et al., 2024) act as powerful modulators of the adult microbiome, thereby influencing mental well-being. Consequently, it has been proposed that microbiome-targeted interventions may impact psychological health in a manner that is strongly shaped by individual lifestyle patterns (Schmidt et al., 2018; Morales-Torres et al., 2023).

Among the interventions known to influence gut microbiome composition and activity, psychobiotics, defined as probiotics that confer mental health benefits, have received substantial scientific attention (Misera et al., 2021; Dinan et al., 2013). Probiotics are reported to have potent positive effects on human mental health, neurodegenerative disorders, mood, and sleep quality (Sivamaruthi et al., 2019; Sivamaruthi et al., 2020; Sivamaruthi et al., 2025; Liu et al., 2025a; Ahmad et al., 2025; Ben Fredj et al., 2026). Clinical studies indicate that combinations of *Lactobacillus* and *Bifidobacterium* strains can exert beneficial effects on stress, anxiety, and broader psychopathological outcomes in healthy populations, as assessed using validated psychological scales (de Le Morvan Sequeira et al., 2022; Messaoudi et al., 2011). Anxiety itself represents a natural adaptive response to perceived threat; however, when excessive or dysregulated, it may progress to clinically significant illness (Gross and Hen, 2004; Krugers and Joëls, 2025). Anxiety disorders, including generalized anxiety disorder, panic disorder with or without agoraphobia, and social anxiety disorder, are among the most prevalent yet underrecognized psychiatric conditions, with treatment typically warranted when symptoms cause marked distress or functional impairment (Bandelow et al., 2017).

Historically, the phenomenology of generalized anxiety disorder has been documented since the late eighteenth century, with earlier diagnostic terms such as “pantophobia” and “anxiety neurosis” used to describe persistent apprehension and episodic panic symptoms (Crocq, 2017). The anxiety disorders constitute one of the leading causes of global disability, ranking among the most prevalent mental health conditions worldwide (Baxter et al., 2014). Epidemiological data indicate that approximately 40 million adults in the United States of America are affected, with a lifetime risk exceeding 40%, underscoring the profound impact of these disorders on quality of life, productivity, healthcare utilization, and socioeconomic burden (Zimmermann et al., 2020).

At the neurobiological level, neurotransmitters such as GABA and serotonin are essential for normal brain function and emotional regulation. Notably, several psychobiotic strains within the *Lactobacillus* and *Bifidobacterium* genera, including *Bifidobacterium dentium, Lactobacillus plantarum,* and *Lactobacillus brevis,* can produce these neuroactive compounds (Schousboe and Waagepetersen, 2007; Barrett et al., 2012; O'Mahony et al., 2015). Additionally, *Lactobacillus plantarum* and *Lactobacillus odontolyticus* have been shown to synthesize acetylcholine, a neurotransmitter involved in regulating diverse cognitive, psychological, and physiological processes (Roshchina, 2016).

**Figure 1:**
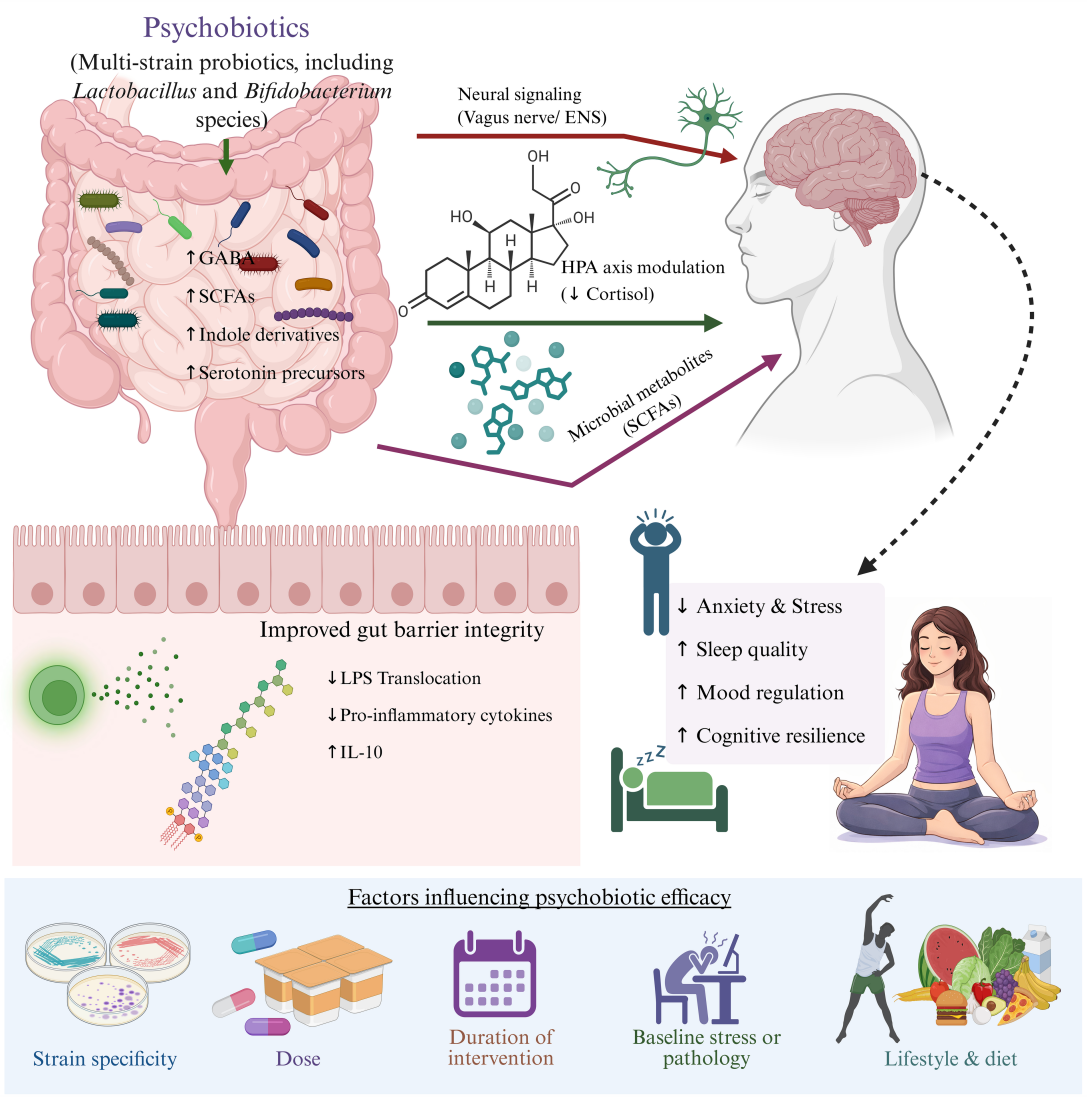
Psychobiotics influence mental health through coordinated neural, immune, endocrine, and metabolic pathways. Specific *Lactobacillus* and *Bifidobacterium* strains modulate gut microbial composition, enhance production of neuroactive metabolites (e.g., GABA, serotonin precursors, short-chain fatty acids, indole derivatives), improve intestinal barrier integrity, and reduce systemic inflammation. These signals are transmitted to the central nervous system via the vagus nerve, hypothalamic–pituitary–adrenal (HPA) axis, and circulating microbial metabolites, ultimately affecting brain regions involved in emotional regulation, stress response, sleep, and cognition. The magnitude of psychobiotic effects is influenced by strain specificity, dosage, baseline psychological state, and lifestyle context. LPS, Lipopolysaccharides; IL-10, Interleukin-10; ENS, Enteric nervous system; GABA, *γ*-aminobutyric acid; SCFAs, Short-chain fatty acids.

Psychobiotics may attenuate anxiety through coordinated modulation of the Amygdala and the hypothalamic–pituitary–adrenal (HPA) axis, two central systems governing emotional reactivity and stress physiology (Cryan et al., 2019). Gut-derived signals influence amygdala activity via multiple mechanisms. Vagal afferent pathways, particularly through the vagus nerve, transmit microbial signals from the intestine to limbic regions, modulating neuronal excitability and emotional processing. The psychobiotic strains, such as *Lactobacillus* and *Bifidobacterium*, can enhance GABA production and regulate tryptophan metabolism, thereby influencing serotonergic and GABAergic signaling pathways that directly control amygdala reactivity. Additionally, microbial metabolites can cross the blood–brain barrier and exert epigenetic and anti-inflammatory effects within limbic structures, reducing microglial activation and dampening stress-related neural sensitization. In parallel, improved gut barrier integrity limits lipopolysaccharide translocation and systemic inflammation, decreasing cytokine-driven activation of the HPA axis and lowering cortisol secretion. Because chronic hyperactivation of the HPA axis and elevated cortisol levels potentiate amygdala hyperresponsiveness and anxiety-like behaviors, microbiota-mediated normalization of endocrine stress signaling may restore emotional regulation. Collectively, these interconnected neural, immune, metabolic, and endocrine mechanisms provide a biologically plausible framework explaining how modulation of the gut microbiota can reduce anxiety by recalibrating both limbic circuitry and stress-axis responsiveness (Figure 1).

In this context, the present review aims to synthesize evidence from clinical trial data only to evaluate the psychobiotic potential of probiotics in modulating anxiety through the gut-brain axis. Specifically, this review seeks to (i) summarize clinical trial findings on probiotic interventions targeting anxiety and related psychological outcomes, (ii) examine strain-specific effects and proposed biological mechanisms underlying psychobiotic action, and (iii) identify key factors influencing therapeutic efficacy, including dosage, participant characteristics, and intervention context, to inform future research and clinical application.

## 2 Methodology

This review was conducted as a structured narrative review focusing on human clinical trials investigating psychobiotics and mental health outcomes via the gut-brain axis. A comprehensive literature search was performed using the electronic databases, such as Scopus, ScienceDirect, Nature, PubMed, Google Scholar and ClinicalTrials.gov. The search covered publications available from 2016 to 2025. The keywords such as psychobiotics, probiotics, gut-brain axis, mental health, clinical trials, depression, anxiety, and human studies were used. Boolean operators (AND, OR) were applied to refine search combinations (e.g., “probiotics AND mental health,” “psychobiotics AND clinical trials,” “gut-brain axis AND depression”).

Studies were included if they were human clinical trials investigating psychobiotics and published in peer-reviewed journals in English. Eligible studies also evaluated mental health outcomes such as depression, anxiety, stress, or cognitive function. Additionally, studies were required to examine mechanisms related to the gut-brain axis.

Studies were excluded if they were animal or *in vitro* studies, or if they were review articles, commentaries, editorials, or conference abstracts. Studies that did not assess mental health–related outcomes or did not involve probiotic or psychobiotic interventions were also excluded. In addition, studies lacking sufficient methodological detail were not included.

Titles and abstracts were screened for relevance. Full texts of potentially eligible studies were then assessed for inclusion based on the criteria described above. Given the narrative nature of this review, database retrieval numbers were not prospectively recorded; therefore, a PRISMA flow diagram is not provided. However, efforts were made to ensure brief coverage of relevant and recent human clinical trials in this field.

## 3 Key findings of recent clinical trials

Clinical trials [Randomized, placebo-controlled clinical trial (RPCT), Randomized, double-blind, placebo-controlled, cross-over trial (RDBPCOT)] provide foundational evidence on the efficacy of psychobiotics in modulating anxiety, stress, and mood-related outcomes by comparing intervention effects directly with a placebo in parallel-group study designs (Table 1).

**Table 1:** Characteristics and outcomes of probiotic clinical trials targeting anxiety.

Study design	Subjects and number	Intervention	Dose	Duration	Key findings	Reference
Effects on healthy subjects						
RDBPCOT	Healthy male volunteers (*n* = 29)	*Lactobacillus rhamnosus* JB-1	1 × 10^9^ CFU	8 weeks	Probiotic was not superior to placebo in modulating anxiety, stress-related behaviors, HPA-axis reactivity, inflammatory markers, and cognitive performance.	Kelly et al. (2017)
RPCT	Healthy adults (*n* = 135).	Cerebiome^®^#	3 billion CFU per day	4 weeks	Probiotic intake interacted significantly with healthy lifestyle behaviors to improve anxiety, emotional regulation, and mindfulness, highlighting the importance of lifestyle factors in psychobiotic effectiveness.	Morales-Torres et al. (2023)
RPCT	Healthy adults (*n* = 30)	*Bifidobacterium breve* M-16 V	20 × 10^9^ cells/day	6 weeks	M-16 V reduced heart rate under stress, increased GABA-like metabolites (pipecolic acid) in stool, and improved mood and sleep in participants with high anxiety, suggesting modulation of the gut-brain axis and autonomic nervous system.	Mutoh et al. (2024)
RPCT	Community-dwelling older adults (*n* = 87)	*Lacticaseibacillus rhamnosus* HN001 (micro-encapsulated vs. non-encapsulated)	5 g maltodextrin and 6 × 10^9^ CFU per sachet; 1 sachet daily with 200 mL milk or water	6 weeks	Encapsulated vs. non-encapsulated probiotics altered visual cortex connectivity and processing speed. Peripheral serotonin was affected. No changes in GABA, glutamate and BDNF.	Rode et al. (2025)
Effects on subjects with stress or anxiety						
RPCT	Healthy college students with anxiety-related symptoms (*n* = 86)	Various multi-strain probiotics	Group 1: 50 billion CFU, 18 species, Group 2: 50 billion CFU, 10 species, Group 3: (placebo): sugar pill, Group 4: 15 billion CFU, 18 species, Group 5: 10 billion CFU, 10 species	28 days	Higher CFU groups showed more effective than species count; probiotics reduced anxiety-related symptoms; ceiling effects were observed in high-distress participants	Tran et al. (2019)
RTPCT	Healthy adults with self-reported psychological stress	Lactoflorene^®^ Plus**	10 mL daily (2 billion CFU/10 mL) for 129 days	2 treatment periods of 45 days with 25-day washout	Probiotic supported mucosal barrier, reduce intestinal inflammation, and improve abdominal discomfort. Increased *Bifidobacterium, Lactobacillus, Faecalibacterium*; decreased *Dialister, Escherichia, Shigella.*	Soldi et al. (2019)
RTPCT	Mild to severe depression subjects (*n* = 71), non-depressed controls (*n* = 20)	Ecologic^®^ BARRIER*	2 g of freeze-dried probiotic powder (daily dose of 1 × 10^10^ CFU), placebo (2 g of freeze-dried maize starch and maltodextrins)	8 weeks	Probiotics improved cognitive patterns associated with depression, but there were no microbiota changes in depressed participants.	Chahwan et al. (2019)
RPRMC	Healthy male students with chronic stress during exam period (*n* = 20)	*Bifidobacterium longum* 1714	1 × 10^9^ CFU/day (with corn starch, magnesium stearate, hypromellose, titanium dioxide)	Two 8 weeks cross over intervention	Improved sleep quality and duration during stress; no effect on cognition or mood.	Moloney et al. (2021)
RPCT	Stressed subjects (*n* = 79)	*Lactobacillus plantarum* P-8	2 g sachet/day (2 × 10^10^ CFU) Fecal samples were collected for metagenomic analysis.	12 weeks	Probiotic modulated gut metagenome, enhanced functional diversity, and was linked to stress/anxiety reduction. Increased microbial diversity; higher SGBs of *Bifidobacterium adolescentis, Bifidobacterium longum*, *Fecalibacterium prausnitzii*; increased neurotransmitter-related metabolites.	Ma et al. (2021)
RPCT	Healthy adults with stress (*n* = 45)	Psychobiotic diet***	Psychobiotic diet	4 weeks	Psychobiotic diet improved perceived stress and induced metabolic changes linked to gut-brain communication. Subtle microbiota changes. Altered fecal lipids and urinary tryptophan metabolites. Microbial volatility associated with stress reduction	Berding et al. (2022)
RPCT	Highly stressed clinical nurses (*n* = 70; PSS ≥ 27)	Heat-killed *Lactobacillus paracasei* PS23 (HK-PS23)	300 mg/day HK-PS23 (equivalent to 10 billion cells)	8 weeks	HK-PS23 significantly reduced blood cortisol compared to placebo and improved anxiety in nurses with higher baseline anxiety.	Wu et al. (2022)
RTPCT	Healthy university students experiencing examination-related stress (*n* = 190)	*Lacticaseibacillus paracasei* Lpc-37	1.56 × 10^10^ CFU/day	10 weeks (8 weeks before examination)	Lpc-37 was safe but did not reduce stress or anxiety; exploratory analyses showed improvements in sleep disturbance and alertness before examinations. No significant changes in fecal microbiota composition	Mäkelä et al. (2023)
RPCT	College students with ADHD (*n* = 67)	*Lactobacillus helveticus, Bifidobacterium animalis* ssp. *lactis, Enterococcus faecium, Bifidobacterium longum, Bacillus subtilis*	8 × 10^9^ CFU per strain	3 months	Probiotic supplementation significantly reduced hyperactivity, improved gastrointestinal symptoms, enhanced academic performance, and showed a negative correlation between FCC and attention/impulsivity.	Levy Schwartz et al. (2024)
RDBPCOT	Moderately stressed healthy adults (*n* = 44)	*Levilactobacillus brevis* P30021 and *Lactiplantibacillus plantarum* P30025	>2 × 10^9^ CFU/day (1:1 mixture)	4 weeks	Probiotics reduced cognitive reactivity to sad mood and rumination, with increased abundance of probiotic taxa in responders but no change in perceived stress.	Casertano et al. (2024)

HPA, Hypothalamic–pituitary–adrenal; CFU, Colony-forming units; PSQI, Pittsburgh Sleep Quality Index; ISI, Insomnia Severity Index; PMS, Premenstrual symptoms; PSS, Perceived stress scale; ADHD, Attention-deficit hyperactivity disorder; FCC, Fingernail cortisol concentrations; SGBs, Species-level genome bins; GABA, γ-aminobutyric acid; BDNF, Brain-derived neurotrophic factor; RPCT, Randomized, placebo-controlled clinical trial; RDBPCOT, Randomized, double-blind, placebo-controlled, cross-over trial; RTPCT, Randomised, triple-blind, placebo-controlled trial; RPRMC, Randomised, placebo-controlled, repeated measures, cross-over; SSRI, Selective serotonin reuptake inhibitors. *Ecologic^®^ BARRIER, *Bifidobacterium bifidum* W23, *Bifidobacterium lactis* W51, *Bifidobacterium lactis* W52, *Lactobacillus acidophilus* W37, *Lactobacillus brevis* W63, *Lactobacillus casei* W56, *Lactobacillus salivarius* W24, *Lactococcus lactis* W19, and *Lactococcus lactis* W58. **Lactobacillus acidophilus LA-5^®^, Bifidobacterium animalis subsp. lactis BB-12^®^, *Lactobacillus paracasei* subsp. paracasei, *Lactobacillus casei* 431^®^, *Bacillus coagulans* BC513, zinc and B vitamins (niacin, B1, B2, B5, B6, B12, and folic acid). ***Psychobiotic diet that influences the microbiota, including whole grains, prebiotic fruits and vegetables, fermented foods, and legumes. #*Lactobacillus helveticus* R0052 and *Bifidobacterium longum* R0175.

### 3.1 Effects on healthy subjects

In an RDBPCOT involving 29 healthy male participants, the impact of *Lactobacillus rhamnosus* JB-1 on stress-related behaviors, physiological responses, inflammation, cognitive function, and brain activity was assessed. Despite promising preclinical findings in animal models, the study found no significant effects of JB-1 on mood, anxiety, sleep quality, or cognitive performance compared to placebo. Additionally, there were no notable changes in inflammatory markers or the HPA stress response. These results underscore the challenges of translating preclinical psychobiotic findings from animal models to human populations and highlight the need for further research in clinical populations, especially those with stress-related disorders (Kelly et al., 2017). The relatively short intervention duration may have limited the detection of longer-term psychobiotic effects. Psychobiotic efficacy depends not only on dose, duration, and microbial colonization but also on the ability of probiotic strains to produce neuroactive metabolites that influence central nervous system signaling. Certain *Lactobacillus* and *Bifidobacterium* species are capable of synthesizing GABA, serotonin precursors, and indole-derived metabolites that modulate neural, immune, and endocrine pathways of gut-brain communication. If the administered strain exhibits limited activity in these metabolic pathways, particularly those influencing GABAergic signaling or tryptophan metabolism, clinical effects on anxiety may be minimal.

The healthy adults were supplemented with *Lactobacillus helveticus* R0052 and *Bifidobacterium longum* R0175. Psychological outcomes, including well-being, quality of life, anxiety, emotional regulation, mindfulness, and interoceptive awareness, were assessed. Although no significant effects of psychobiotic supplementation were observed across the full sample, healthy lifestyle behaviors were consistently associated with improved well-being outcomes. Notably, interaction analyses revealed that psychobiotic intake produced beneficial post-treatment effects on anxiety, emotional regulation, and mindfulness only among participants with higher adherence to healthy behaviors. These findings suggest that psychobiotic efficacy may depend on broader lifestyle context rather than probiotic intake alone. Overall, the study underscores the importance of accounting for lifestyle variables in psychobiotic and mental health research and supports an embodied, bottom-up framework for understanding psychological functioning (Morales-Torres et al., 2023).

*Bifidobacterium breve* M-16 V has been investigated as a psychobiotic with potential effects on mood, sleep, and autonomic nervous system regulation. The supplementation of M-16 V was well tolerated and compliant; participants with higher baseline anxiety demonstrated improvements in mood and sleep scores. Supplementation also decreased stress-related heart rate responses and increased fecal pipecolic acid levels, suggesting modulation of the gut-brain axis. These findings indicate that M-16 V may influence autonomic nervous system balance and gut-derived neuromodulators, potentially contributing to enhanced mood and sleep in individuals experiencing elevated anxiety. The study supports further exploration of this psychobiotic for stress-related outcomes, particularly in populations with higher baseline anxiety or mood vulnerability (Mutoh et al., 2024).

The effects of micro-encapsulated and non-encapsulated *Lacticaseibacillus rhamnosus* HN001 in community-dwelling individuals on brain health and cognitive function were recently reported. Resting-state functional magnetic resonance imaging revealed significant differences in functional connectivity within regions associated with visual processing and perception between the two probiotic formulations, although no changes in brain morphometry were observed. Secondary outcomes included magnetic resonance spectroscopy, quantification of GABA and glutamate, as well as peripheral markers, such as serotonin and brain-derived neurotrophic factor (BDNF). While peripheral serotonin levels showed significant changes over time, no notable differences were detected in GABA, glutamate, or BDNF. Cognitive testing indicated significant improvements in processing speed, whereas other domains, including short-term memory, anxiety, depression, perceived stress, and sleep quality, remained largely unaffected. The study design, which ranked participants by sex and age and employed high compliance and quality control in neuroimaging data acquisition, provided robust insight into psychobiotic effects on brain function. Micro-encapsulation appeared to influence gut delivery and, consequently, the impact on functional brain connectivity. Limitations included potential learning effects on repeated cognitive tests despite the use of alternative test versions, as well as the possibility of random fluctuations influencing specific test outcomes. Nonetheless, the study demonstrates that targeted psychobiotic interventions can modulate functional connectivity in the aging brain and selectively improve cognitive performance. These findings underscore the importance of considering psychobiotic formulation and delivery methods in designing interventions for older adults, offering promising avenues for supporting cognitive health through gut-brain axis modulation in healthy aging populations (Rode et al., 2025).

Rather than producing broad psychological improvements, psychobiotics may influence selective neurobiological or cognitive domains. JB-1 showed no effect on mood or HPA activity, yet HN001 modulated functional brain connectivity and processing speed without affecting emotional outcomes. Similarly, M-16 V altered autonomic responses and gut-derived metabolites. This suggests that psychobiotics may first alter neural circuitry or physiological regulation before producing detectable subjective mood changes.

### 3.2 Effects on subjects with stress or anxiety

In an RPCT, 86 healthy college students were randomized and separated into five groups. Group 1 received high CFU, and high species count psychobiotics (50 billion CFU, 18 species), Group 2 received high CFU but low species count (50 billion CFU, 10 species), Group 3 was the placebo control, Group 4 received low CFU, and high species count psychobiotics (15 billion CFU, 18 species), and Group 5 received low CFU, and low species count psychobiotics (10 billion CFU, 10 species). After 28 days of daily intake, participants completed an exit survey assessing anxiety and related outcomes. The study found that psychobiotics improved panic and neurophysiological anxiety, negative affect, worry, and negative mood regulation. *Post hoc* analyses indicated that CFU levels were more strongly associated with improvements than species counts. Interestingly, participants with higher baseline distress reported greater improvements, suggesting a ceiling effect. These findings provide preliminary evidence that psychobiotics have therapeutic potential in reducing anxiety and highlight the importance of both CFU and species composition in determining efficacy. While the study has several limitations, it offers promising directions for future research, suggesting that not all probiotics are equally effective and emphasizing the need for further investigation into psychobiotics as a mental health intervention (Tran et al., 2019).

A study aimed to evaluate whether a psychobiotic product, Lactoflorene^®^ Plus, could prevent alterations in the immune response linked to self-reported stress and microbiota composition. Healthy adult volunteers experiencing psychological stress were enrolled and randomly assigned to either a placebo or a probiotic group. Various salivary stress markers (*α*-amylase, cortisol, chromogranin A), immunological parameters (sIgA, NK cell activity, IL-8, IL-10, TNF-α) in feces, and intestinal microbiota composition were assessed. Although the psychobiotic product did not directly impact salivary stress markers or NK cell activity, it did reduce abdominal pain, enhance fecal sIgA levels, and increase IL-10 production. The probiotic product also induced a moderate increase in beneficial bacteria such as *Bifidobacterium* and *Lactobacillus* spp. and *Faecalibacterium* spp., while decreasing populations of potentially harmful bacteria, including *Dialister* spp., *Escherichia*, and *Shigella*. The administration of Lactoflorene^®^ Plus helped to support the mucosal barrier by promoting short-chain fatty acid producers and decreasing intestinal inflammation, which, in turn, alleviated abdominal discomfort. The study’s results suggest that the psychobiotic intake indirectly supported the mucosal barrier, reducing intestinal inflammation and providing relief from abdominal discomfort and common stress-related symptoms. This protection may help explain the reduction in stress-related conditions and the broader influence of psychobiotics on physical health. Additionally, the anti-inflammatory and immunological effects of the psychobiotics may play a role in alleviating mood disturbances, given the well-established link between pro-inflammatory cytokines and neuropsychological symptoms (Soldi et al., 2019).

In a clinical trial investigating the effect of the psychobiotic supplement Ecologic^®^ BARRIER on depressive symptoms, participants assigned to the probiotic group were provided with two sachets daily, each containing 2 g of freeze-dried psychobiotic powder. This provided a total daily dose of 1 × 10^10^ CFU, with each sachet containing 2.5 × 10^9^ CFU of probiotics. The trial aimed to explore the impact of these probiotics on depressive symptoms in individuals with mild to severe depression. Seventy-one participants were randomly assigned to receive either the psychobiotic or a placebo, with the intervention lasting for eight weeks. Pre- and post-intervention measures of depressive symptoms, vulnerability markers, and gut microbiota composition were taken, and the results were compared to a non-depressed group. While all participants showed some improvement, those in the psychobiotic group experienced a significantly greater reduction in cognitive reactivity, particularly in those with mild to moderate depression. However, probiotics did not significantly alter the microbiota composition in the depressed participants, though a correlation was found between *Ruminococcus gnavus* and one depression metric. Despite a high attrition rate and the need for more specific microbiota analysis, the study suggests that psychobiotics can positively affect psychological variables related to depression susceptibility. Future research should focus on more sensitive measures of physiological stress, such as cortisol levels, as well as investigating optimal dosages, timeframes, and specific gut microbiota strains. This study offers evidence supporting the use of probiotics as a potential adjunct to existing treatments like cognitive behavioral therapy, promoting a holistic approach that integrates gut health into mental health management (Chahwan et al., 2019).

Targeting the gut microbiome as a therapeutic strategy for psychological disorders has gained increasing attention in recent years. Variation in microbiota composition and restoration of a stable microbiome using targeted interventions, often referred to as psychobiotics, including Bifidobacteria, has shown promise in preclinical studies. However, human data on the potential health benefits of these probiotics remains limited. *Bifidobacterium longum* 1714 has been shown to reduce the effects of acute stress in humans, yet its impact under prolonged stress conditions has not been explored. To address this, a randomized, placebo-controlled, repeated-measures, crossover study was conducted in 20 male university students to examine the effects of *Bifidobacterium longum* 1714 on stress, cognitive performance, and mood. The intervention coincided with the university exam period, used as a naturalistic model of chronic stress. Participants were assessed using self-reported measures of stress, depression, sleep quality, physical activity, gastrointestinal symptoms, cognition, and mood, alongside cognitive testing via the Cambridge Neuropsychological Test Automated Battery. During the exam period, stress and depression scores increased in both the probiotic and placebo groups. While *Bifidobacterium longum* 1714 supplementation did not improve measures of working memory, visual memory, sustained attention, or perception compared with placebo, it did significantly improve overall sleep quality and duration during exam stress. These findings indicate that although *Bifidobacterium longum* 1714 may have specific beneficial effects on sleep during prolonged stress. This supports the broader concept that probiotics can modulate aspects of brain health, even if their effects are strain- and context-specific. Further mechanistic studies are warranted to understand how *Bifidobacterium longum* 1714 influences sleep regulation under chronic stress conditions and to determine the precise clinical applications of psychobiotics in mental health (Moloney et al., 2021).

Stress has been shown to disrupt the balance of the human intestinal microbiota, which can contribute to mental health problems such as anxiety and depression. The supplementation of *Lactobacillus plantarum* P-8 for 12 weeks alleviated stress and anxiety in adults. Participants received daily oral supplementation of *Lactobacillus plantarum* P-8 or a placebo for 12 weeks. In-depth analysis of the fecal metagenomes revealed that the placebo group exhibited a significantly greater shift in gut microbial composition over the 12 weeks, as measured by Aitchison distance, along with a significant decrease in microbial diversity, measured by the Shannon index. In contrast, these changes were not observed in the probiotic group. Moreover, the probiotic group showed significant enrichment of species-level genome bins (SGBs) associated with *Bifidobacterium adolescentis, Bifidobacterium longum,* and *Faecalibacterium prausnitzii*, while SGBs representing *Roseburia faecis* and *Fusicatenibacter saccharivorans* decreased. The 12-week supplementation also enhanced the diversity of neurotransmitter-synthesizing and consuming SGBs and increased predicted levels of neuroactive microbial metabolites, including short-chain fatty acids, GABA, arachidonic acid, and sphingomyelin. These findings suggest a potential link between probiotic-induced modulation of the gut microbiota and alleviation of stress and anxiety, supporting the involvement of the gut-brain axis in relieving stress-related symptoms. Importantly, the beneficial effects appeared to rely not only on maintaining microbial diversity but also on specific metagenomic changes at the SGB and functional gene levels, highlighting the complex mechanisms through which probiotics can influence mental health (Ma et al., 2021).

The impact of diet on microbiota composition and its role in supporting optimal mental health have garnered significant attention. However, the potential of whole dietary approaches to exert psychobiotic effects remains largely understudied. Berding et al. investigated the influence of a psychobiotic diet rich in prebiotic and fermented foods on microbial profiles, gut function, and mental health outcomes in a healthy human population. Forty-five adults were randomly assigned to either a psychobiotic diet or a control diet for 4 weeks. The psychobiotic diet resulted in a 32% reduction in perceived stress compared to 17% in the control group, although the differences between groups were not statistically significant. Despite this, individuals with higher adherence to the psychobiotic diet experienced more substantial reductions in perceived stress. While the dietary intervention induced only subtle changes in microbial composition and function, it significantly altered the levels of fecal lipids and urinary tryptophan metabolites. Notably, increased microbial volatility was associated with greater changes in perceived stress scores, suggesting a link between microbial shifts and stress alleviation. Although the diet did not produce significant differences in anxiety levels, the intervention did show promise in improving perceived stress in the psychobiotic group. These findings indicate that microbiota-targeted dietary approaches hold potential for modulating gut-brain communication and reducing perceived stress (Berding et al., 2022).

Occupational stress among nurses is associated with dysregulation of the HPA axis, heightened glucocorticoid secretion, and inflammatory responses, all of which may contribute to adverse mental and physical health outcomes. Highly stressed female clinical nurses received either heat-killed *Lactobacillus paracasei* PS23 or placebo for eight weeks. Although both groups showed improvements across multiple psychological and behavioral measures over time, only participants receiving the psychobiotic intervention exhibited a significant reduction in circulating cortisol levels. Notably, among nurses with elevated baseline anxiety, psychobiotic supplementation was associated with significant improvements in anxiety symptoms compared with placebo. The intervention was well tolerated, with no safety concerns reported. These findings suggest that heat-killed psychobiotics may exert stress- and anxiety-reducing effects, potentially via modulation of stress hormone regulation, and support further investigation into microbiome-related mechanisms underlying stress resilience in high-risk occupational populations (Wu et al., 2022).

*Lacticaseibacillus paracasei* Lpc-37 has previously been proposed as a psychobiotic with stress-reducing potential; however, evidence from well-controlled trials remains mixed. The ChillEx study evaluated the effects of daily Lpc-37 supplementation in healthy university students exposed to chronic academic stress during examinations. Lpc-37 did not significantly reduce state anxiety before examinations compared with placebo, nor did it demonstrate robust effects on secondary psychological outcomes after correction for multiple testing. Exploratory analyses suggested modest improvements in certain sleep-related parameters and alertness measures in the probiotic group. Fecal microbiota analyses revealed no differences in microbial diversity or composition between groups, indicating that Lpc-37 did not induce detectable shifts in gut microbial structure. The intervention was well tolerated, with no safety concerns identified. Notably, the absence of a stress increase in the study population before examinations may have limited the ability to detect treatment effects. Overall, these findings underscore the importance of appropriate population selection, stress induction models, and functional microbiome assessments when evaluating psychobiotics, and they highlight the challenges of translating promising preclinical data into clinical benefit in healthy, low-symptom populations (Mäkelä et al., 2023).

Attention deficit hyperactivity disorder (ADHD) is a prevalent neuropsychiatric condition often persisting into adulthood, characterized by inattention, impulsivity, and hyperactivity. Emerging evidence suggests that modulation of the gut microbiota via probiotics may influence ADHD symptoms. College students with ADHD were administered a multi-strain psychobiotic supplement or placebo daily for 3 months. ADHD-related outcomes were evaluated using computerized performance tests, standardized questionnaires, and academic performance records, while long-term HPA axis activity was measured via fingernail cortisol concentrations. The study found that probiotic supplementation significantly reduced hyperactivity, improved gastrointestinal symptoms, and enhanced academic performance. Subgroup analyses indicated that younger participants derived greater benefit, and lower cortisol levels were correlated with improved attention and impulse control (Levy Schwartz et al., 2024).

*Levilactobacillus brevis* P30021 and *Lactiplantibacillus plantarum* P30025 can produce GABA and acetylcholine. A RDBPCOT then evaluated the cognitive and psychological effects of this probiotic combination in moderately stressed but otherwise healthy adults. Despite no measurable effects on perceived stress, supplementation was associated with significant improvements in depressive cognitive patterns, including reduced cognitive reactivity to negative mood and decreased rumination. These changes suggest a potential benefit in mitigating maladaptive cognitive processes linked to depression rather than directly lowering stress levels. Microbiota analyses indicated increased abundance of the administered probiotic genera among treatment responders, supporting successful gut colonization, although no clear associations were observed between fecal neurotransmitter concentrations and psychological outcomes. The intervention was well tolerated and safe; however, effects may have been influenced by co-administered micronutrients, such as vitamin D. Overall, these findings highlight the domain-specific effects of psychobiotics on mood-related cognition in healthy individuals and underscore the need for larger, longer-term studies in clinically stressed populations, with greater consideration of biological heterogeneity, functional microbiome outputs, and neurophysiological endpoints (Casertano et al., 2024).

Across these trials, psychobiotic effects appear strain-specific, domain-specific, and population-dependent, rather than uniformly anxiolytic or antidepressant. Multi-strain, high-CFU formulations (Tran et al., 2019) showed broader reductions in anxiety-related symptoms, with CFU load emerging as more influential than species diversity, and greater benefits observed in participants with higher baseline distress. In contrast, several studies in healthy or low-symptom populations (Moloney et al., 2021; Mäkelä et al., 2023) reported limited effects on primary stress or anxiety outcomes, although selective benefits were observed in alertness, highlighting context sensitivity and symptom severity as moderators of response.

Mechanistically, effects often occurred without large-scale shifts in overall microbial diversity, but rather through functional or immunological modulation. For example, Soldi et al. (2019) and Wu et al. (2022) demonstrated changes in mucosal immunity (↑sIgA and IL-10) and cortisol regulation, while Ma et al. (2021) and Liu et al. (2025b) identified enrichment of neuroactive metabolite pathways (e.g., GABA, Short-chain fatty acids) and specific taxa-level changes rather than global microbiome restructuring. These findings suggest that functional metagenomic shifts and host immune-endocrine modulation may be more critical than compositional diversity.

Several studies also indicate that psychobiotics may preferentially influence cognitive-affective processing rather than subjective stress ratings. Casertano et al. (2024) and Chahwan et al. (2019) reported reductions in maladaptive cognitive reactivity and rumination without broad mood changes, while Levy Schwartz et al. (2024) observed improvements in ADHD-related hyperactivity and academic performance alongside reduced cortisol. This pattern supports a bottom-up neurocognitive modulation framework, where gut interventions alter stress reactivity, executive function, or sleep regulation before affecting global mood states.

Importantly, delivery form and intervention duration appear relevant. High-dose formulations, longer supplementation periods (8 to 12 weeks), and targeted strains with known neuroactive potential (e.g., GABA producers such as BLa80 or P30021/P30025) showed more consistent functional outcomes compared to short-term or low-dose interventions. Diet-based psychobiotic approaches (Berding et al., 2022) further suggest that whole-diet modulation may exert subtler but systemic effects, potentially interacting with adherence and baseline microbiome volatility.

Collectively, these studies support a precision psychobiotic model: efficacy depends on CFU dose, strain functionality, delivery matrix, host baseline vulnerability, stress context, and outcome domain measured. Rather than universal mood enhancers, psychobiotics appear to act as modulators of stress physiology, sleep regulation, immune signaling, and cognitive-emotional processing, highlighting the need for targeted, phenotype-driven clinical applications.

## 4 Strain-specific psychobiotic mechanisms

These mechanistic findings provide a framework for interpreting the variability observed in clinical trials and suggest that successful psychobiotic interventions may depend on demonstrable engagement of specific neuroactive pathways. Recent research has begun to elucidate the molecular mechanisms underlying the antidepressant effects of psychobiotics, highlighting gut-derived metabolites as key mediators. In preclinical and clinical investigations, supplementation with *Bifidobacterium breve* and related strains was shown to increase indole-3-lactic acid (ILA) levels, a metabolite capable of modulating neuroinflammation through aryl hydrocarbon receptor (AhR) signaling. In depressed mice, hippocampal ILA levels were reduced, a deficit reversed by administration of ILA-producing bifidobacteria, with corresponding improvements in depressive-like behaviors. Mechanistic studies identified the aromatic lactate dehydrogenase (Aldh) gene as critical for ILA biosynthesis, and strains lacking Aldh failed to produce antidepressant effects, indicating a genomic and metabolic basis for strain-specific psychobiotic activity. Human studies further supported these findings, demonstrating increased circulating ILA following *Bifidobacterium breve* supplementation in both depressed and healthy adults. These results suggest that the antidepressant potential of psychobiotics may depend on their capacity to produce specific neuroactive metabolites, with *Bifidobacterium* strains harboring Aldh emerging as promising candidates for targeted microbial therapies. Limitations include species-level rather than strain-level differentiation of Aldh-positive bifidobacteria, small sample sizes in animal studies, and incomplete evaluation of sex-specific effects, underscoring the need for larger, longer-term clinical trials and functional metabolomics analyses to fully characterize ILA-mediated modulation of the gut-brain axis (Qian et al., 2024).

Secondary analyses provide deeper insights into the nuanced effects of probiotics and gut microbiota on mental health, allowing for exploration of biomarkers, mechanistic pathways, and precision-targeted interventions. In a secondary analysis of an RPCT involving adults with major depressive disorder, baseline fecal microbiota composition was examined in relation to remission status after 12 weeks of treatment. While overall microbial diversity did not differ between remitters and non-remitters, specific bacterial genera at baseline were strongly associated with clinical outcome. A machine-learning model using a limited set of microbial genera demonstrated high accuracy in predicting remission, with greater relative abundance of short-chain fatty acid-producing taxa such as Faecalibacterium, Agathobacter, and Roseburia linked to favorable treatment response. Longitudinal analyses further showed that significant shifts in gut microbial composition occurred only among individuals who achieved remission. These findings highlight the potential of the gut microbiome as a biomarker for antidepressant response in geriatric depression and support the emerging concept of microbiome-informed precision psychiatry, including the future development of microbiota-based adjunctive interventions (Lee et al., 2022).

Dysbiosis has increasingly been recognized as a key factor in the pathophysiology of mood disorders, with short-chain fatty acid metabolites produced by bacterial fermentation in the colon playing a critical role in gut-brain communication. Probiotics have shown promise in alleviating depressive symptoms, particularly when used as an adjunct to standard antidepressant therapy. In a secondary analysis of a two-arm, parallel-group, RPCT, the effects of a probiotic formulation containing *Lactobacillus helveticus* Rosell^®^-52 and *Bifidobacterium longum* Rosell^®^-175 (R0052/R0175) on fecal short-chain fatty acid levels were evaluated in participants with depression over a 60-day intervention period. Stratification was performed based on participants’ baseline antidepressant medications to explore potential interactions. Fecal short-chain fatty acids were measured using gas chromatography, and pre-intervention clinical, socio-demographic, and laboratory data were assessed. The results demonstrated that probiotic supplementation significantly decreased fecal isovaleric acid levels compared with placebo when administered alongside non-selective serotonin reuptake inhibitors (non-SSRIs), with a large effect size, while no significant effects were observed when combined with SSRIs or when used alone. Notably, decreases in isovalerate levels corresponded with clinical improvement in depressive symptoms in the probiotic plus non-SSRI group, suggesting a potential mechanistic link between short-chain fatty acids modulation and antidepressant efficacy. These findings indicate that R0052/R0175 may enhance the antidepressant action of non-SSRIs partly through interactions with isovaleric acid, highlighting a possible role for gut microbiota metabolites as mediators of psychotropic effects (Gawlik-Kotelnicka et al., 2025).

Taken together, current evidence suggests that psychobiotic effects are highly strain-specific and influenced by individual context, with more pronounced benefits in populations with elevated baseline stress or mood vulnerability. Reported outcomes include improvements in mood, cognitive patterns, sleep quality, and neurophysiological regulation; however, findings are inconsistent, particularly among healthy or low-stress individuals. Variations in gut microbiota composition and microbial metabolites, including short-chain fatty acids, GABA, serotonin precursors, and indole-derived metabolites such as ILA, may partly explain differential stress and antidepressant responses observed across clinical trials.

## 5 Future perspectives

Future research should prioritize larger, adequately powered trials with diverse populations, including individuals with clinical anxiety, depression, or stress-related disorders, to establish reproducible efficacy (Butler et al., 2025). Integration of multi-omics approaches, including metagenomics, metabolomics, and functional neuroimaging, will be critical to elucidate mechanisms linking specific microbial strains to neuroactive metabolites, short-chain fatty acids, and neurotransmitter modulation. Personalized psychobiotic interventions based on baseline microbiota composition, neurochemical profiles, and symptom severity may enhance clinical outcomes. Further exploration of psychobiotic formulation, encapsulation techniques, dosage, and duration will optimize gut delivery and therapeutic impact. Additionally, combining probiotics with lifestyle interventions, dietary strategies, or conventional therapies such as cognitive behavioral therapy may provide synergistic benefits. Ultimately, advances in precision microbiome-based psychiatry could enable targeted modulation of the gut-brain axis to improve mental health outcomes, offering a novel, holistic approach to anxiety, stress, and mood disorders.

## 6 Conclusion

Clinical evidence indicates that probiotics can exert measurable effects on psychological outcomes through modulation of the gut-brain axis, with benefits most pronounced under conditions of elevated stress, subclinical mood disturbances, or in combination with standard therapies. Specific strains, such as *Bifidobacterium longum* and *Lactobacillus helveticus*, have demonstrated reductions in depressive symptoms, cognitive reactivity, and stress-related physiological markers, while others, including *Lactobacillus rhamnosus* and *Lacticaseibacillus paracasei*, showed more selective effects on sleep, anxiety, or neuroendocrine responses. Mechanistic analyses reveal that psychobiotic effects may be mediated via modulation of gut microbial composition, short-chain fatty acid production, neurotransmitter synthesis, and systemic anti-inflammatory pathways. However, variability in outcomes across trials emphasizes the influence of participant characteristics, baseline psychological state, dosage, strain specificity, and intervention duration on efficacy. Overall, psychobiotics represent a promising adjunctive strategy for mental health interventions, particularly for stress management, anxiety reduction, and mood enhancement, although their application must be tailored to individual needs and clinical contexts.
